# Recyclable Multifunctional Ionic Liquids for Sustainable Electroorganic Oxidations

**DOI:** 10.1021/acselectrochem.5c00108

**Published:** 2025-07-09

**Authors:** Astrid E. Delorme, Andrew Jordan, Jessica Streets, Victor Sans, Stephen P. Argent, Helen F. Sneddon, Peter Licence, Darren A. Walsh

**Affiliations:** † School of Chemistry, 6123 University of Nottingham, University Park, Nottingham NG7 2RD, UK; ‡ GSK Carbon Neutral Laboratories for Sustainable Chemistry, University of Nottingham, Jubilee Campus, Nottingham NG7 2TU, UK; § Faculty of Engineering, University of Nottinghamm, University Park, Nottingham NG7 2RD, UK; ∥ Institute of Advanced Materials (INAM), 16748 Universitat Jaume I, Avda. Sos Bainat s/n, 12071, Castellon, Spain; ⊥ Department of Chemistry, University of York, Heslington, York YO10 5DD, UK; ¶ Green Chemistry, GSK, Medicines Research Centre, Gunnels Wood Road, Stevenage, Hertfordshire SG1 2NY, UK

**Keywords:** Electrosynthesis, TEMPO, Ionic Liquid, Voltammetry

## Abstract

The study of electrochemical oxidations has wide-ranging implications, from the development of new electrocatalysts for fuel cells for energy conversion, to the synthesis of fine chemicals. 2,2,6,6-Tetramethylpiperidine-1-oxyl (TEMPO) has been used for decades as a sustainable, metal-free mediator for chemical oxidations and is now being used for electrochemical oxidations. We describe here a novel approach to TEMPO-mediated electrooxidations, in which the chemical input and waste generated during electrooxidations of alcohols are minimized by using a multifunctional room temperature ionic liquid (RTIL) to facilitate flow electrosynthesis. Our three-pronged approach involves the following: (1) the use of a recyclable alkylimidazolium-based RTIL to replace the electrolytes and volatile organic solvents typically used in TEMPO-mediated alcohol electrooxidations; (2) pairing alcohol oxidation at the positive electrode with electroreduction of the cationic component of the RTIL at the negative electrode to generate the base required for the electrosynthesis; (3) incorporation of the catalytic TEMPO moiety in our RTIL-based system. We demonstrate the possibilities offered by our strategy using a range of alcohols, illustrating the scope of reactions that can be driven using our strategy and demonstrating the opportunities offered by task-specific, multifunctional RTILs for electroorganic synthesis.

## Graphical abstract

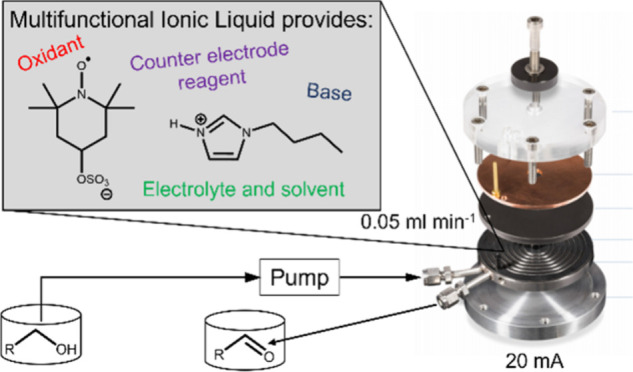

## Introduction

Increasing interest in the development of sustainable chemical processes has led to renewed interest in electroorganic synthesis in recent years.^1–3^ Provided that waste is minimized, yields are high, and the required apparatus and materials are accessible and inexpensive, electrosynthesis powered by renewable energy could provide sustainable and accessible routes to a range of functionalities.^4^ The uptake of electrosynthetic techniques is also being facilitated by the growing range of equipment available for organic electrosynthesis, which includes batch,^5–7^ flow,^8,9^ and even wireless systems.^10^

Increased activity in electrosynthesis is also driving research into the development of new solvents and additives for electrosynthesis. The use of redox mediators that can shuttle electrons between electrodes and reagents (so-called indirect electrosynthesis) can improve chemoselectivity and enhance reaction rates.^1,11,12^ Nitroxyl radicals, such as 2,2,6,6-tetramethylpiperidine-1-oxyl (TEMPO), which can be used as a relatively sustainable catalyst for oxidations,^13–15^ are now being used in electrooxidations.^16,17^ In terms of new solvents for electrosynthesis, RTILs are becoming more common in electrochemistry, as they can act as both solvents and electrolytes, are electrochemically and chemically stable, and can dissolve a wide range of solutes.^18–20^ The ever-expanding range of viable structures of RTILs also means that the properties of RTILs can often be tuned for specific applications.^21^

TEMPO-mediated oxidation of alcohols is represented in Scheme 1, showing the formation of the active oxonium species. Regeneration of this species can be facilitated by a co-catalyst such as NaNO_2_ ^22^ or HOCl,^23^ or electrochemically.^24,25^ The process has been conducted in organic solvents and in RTIL-based media.^25,26^ A significant advantage of electrochemical methods is that they do not require co-catalysts, but the need for a stoichiometric base remains a shortcoming with respect to waste minimization and atom efficiency. Moreover, recycling of TEMPO is challenging.^27^ RTIL-supported TEMPO, where R in Scheme 1 includes a cationic component, has also been used for oxidation of alcohols using a variety of co-catalysts, and its use can potentially facilitate recycling of the TEMPO moiety.^22,27–30^

**Scheme 1:**
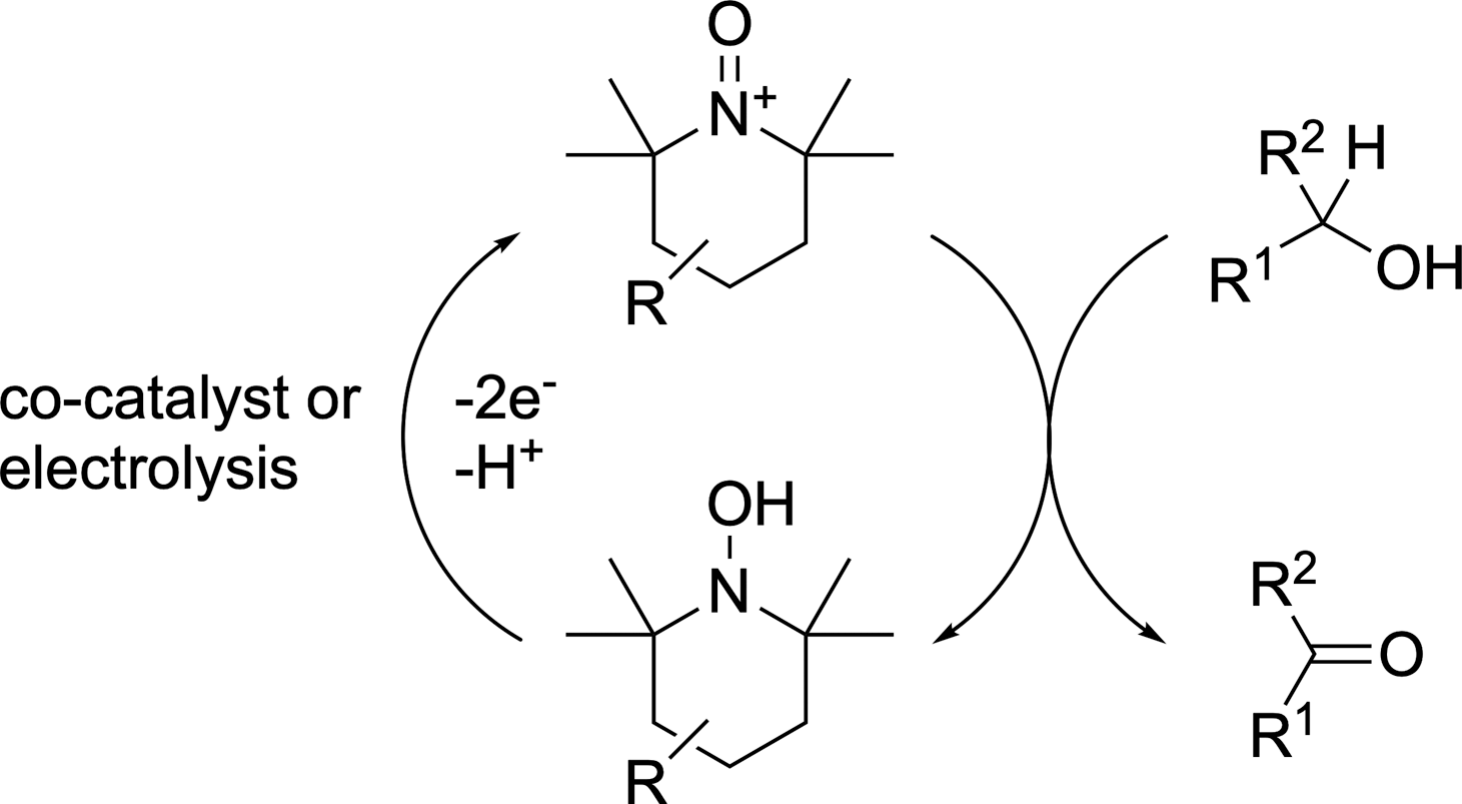
TEMPO-Mediated Alcohol Oxidation^a^

a Regeneration of the oxonium active species can be facilitated by a co-catalyst or electrolysis.

In this contribution, we describe an electrosynthetic strategy that exploits the advantages offered by multifunctional RTILs as solvents for electrochemistry and flow electrosynthesis.^31–33^ The first part of our strategy exploits the ability to form TEMPO-based RTILs, the reactivity of which can rival those of homogeneous TEMPO systems^22,27,28,34^ and which brings the advantages of RTILs, including tunable reactivity and recyclability, to the oxidation catalyst.^27,35^ To the best of our knowledge, the synthesis and characterization of 1-butylimidazolium TEMPO-4-sulfate, [C_4_HIm]­[(TEMPO)­OSO_3_] has not yet been reported, and its characterization data including single-crystal X-ray structure is described in the SI.

During electrolysis, [C_4_HIm]^+^ cations are reduced at the negative electrode to yield 1-butylimidazole (C_4_Im) and H_2_ (Scheme 2 step 1), both of which are advantageous to the flow electrosynthesis; C_4_Im is necessary to deprotonate the alcohol (Scheme 2 Step 2), while evolution of H_2_ bubbles can improve cell performance in microfluidic channels due to increased turbulence and mass transfer to the electrodes.^36^ *In-situ* generation of basic species at negative electrodes is a common feature of synthetic electrooxidations.^36,37^ Our approach capitalizes on this aspect in a novel way by integrating it into a recyclable RTIL system in a flow-through setup. The next part of our electrosynthetic cycle involves electrochemical oxidation of [C_4_HIm]­[(TEMPO)­OSO_3_] at the positive electrode. This step generates the oxonium species that drives oxidation of the target reagent (Scheme 2 Steps 3 and 4) and TEMPO-hydroxylamine species, which in turn reforms the protic RTIL cations (Scheme 2 Step 5). As we discuss below, our flow process results in high yields during the oxidation of a range of alcohols, including primary, secondary, aromatic and aliphatic alcohols, containing different functional groups.

**Scheme 2:**
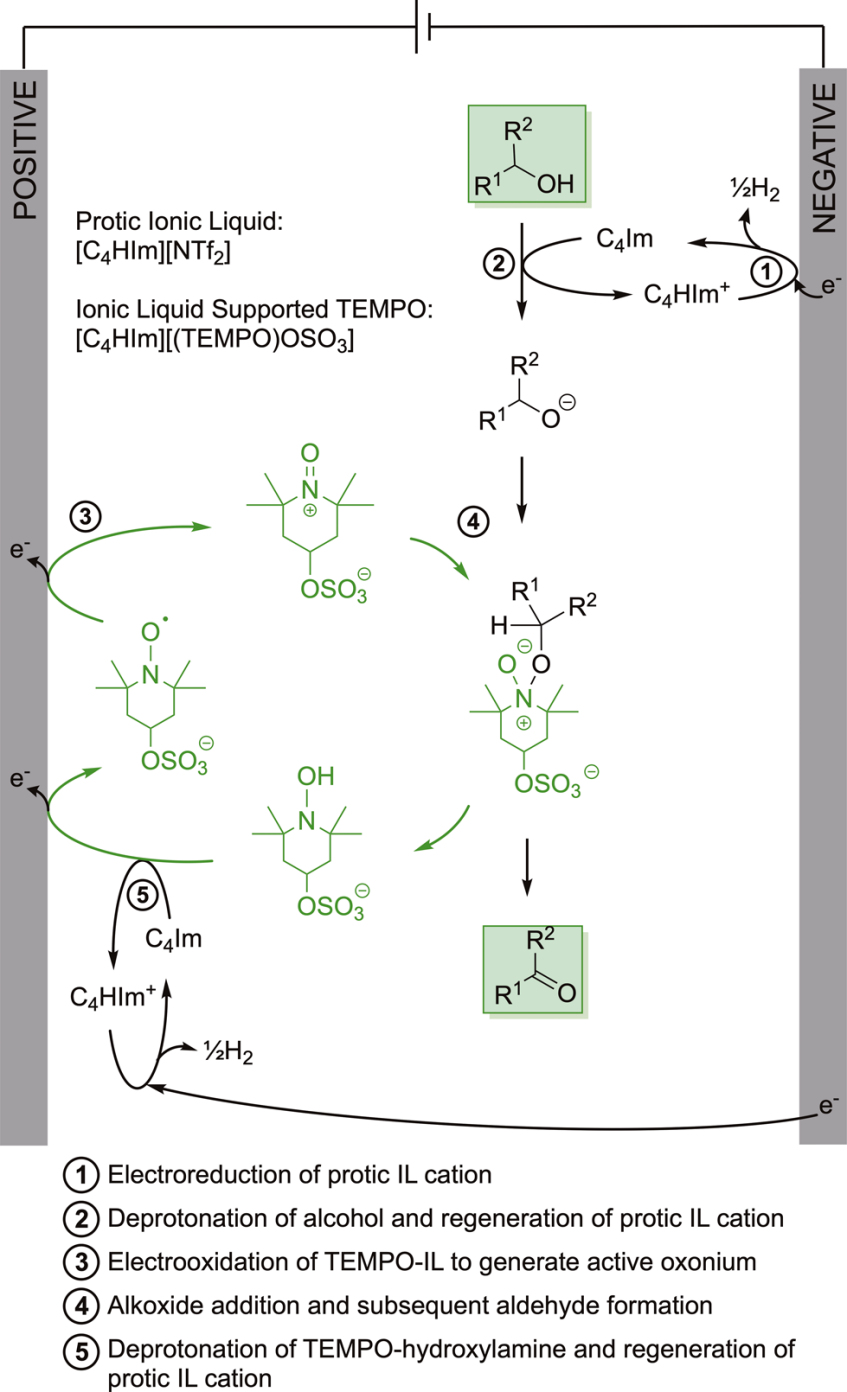
Proposed Alcohol Electrooxidation Mechanism, Which Pairs Electrogeneration of the Active Oxonium Species at the Positive Electrode with Electrogeneration of the Base Required at the Negative Electrode

## Experimental Methods

### Cyclic Voltammetry

Cyclic voltammograms were recorded using an Autolab PGSTAT302 (EcoChemie, the Netherlands). A three-electrode system, consisting of a 3 mm diameter glassy carbon (GC) disk or 2 mm diameter Pt disk working electrode (WE), Pt flag counter electrode (CE), and Ag wire as a quasi-reference electrode (QRE), was used. Prior to use, the GC and Pt electrodes were polished using aqueous suspensions of 0.05 μm alumina and rinsed thoroughly with H_2_O. All solutions were deoxygenated prior to use by bubbling with Ar for at least 10 min. Compensation for uncompensated ohmic resistance was performed during each measurement using positive-feedback correction.

### Flow Electrolysis

Flow electrolysis was performed using an Ammonite8 cell (Figure S2) from Cambridge Reactor Design (Cambridgeshire, UK). 0.2 mol dm^–3^ alcohol, 0.06 mol dm^–3^ [C_4_HIm]­[(TEMPO)­OSO_3_], and 0.2 mol dm^–3^ tertbutylbenzene (as the internal standard for monitoring using gas chromatography-mass spectrometry) were dissolved in 2.5 cm^3^ [C_4_HIm]­[NTf_2_]. Initially, 1.0 cm^3^ of [C_4_HIm]­[NTf_2_] was pumped through the cell, and the reaction solution was then pumped through the cell at 0.05 cm^3^ min^–1^. Electrolysis was carried out under constant current of 20 mA. After the initial 1 cm^3^ of [C_4_HIm]­[NTf_2_] had passed through the cell, the products were collected in a sample vial at the outlet of the cell. Products were extracted from the RTIL with toluene and analyzed using NMR spectroscopy and gas chromatography.

## Results and Discussion

### Reduction of Protonated Base in TEMPO-Mediated Alcohol Oxidations

In conventional TEMPO-mediated alcohol oxidations, oxidation of TEMPO occurs at the positive electrode, while reduction of the supporting electrolyte and/or solvent occurs at the negative electrode. Our strategy instead involves reduction of the protic cations of an RTIL at the negative electrode, to generate the base required for the electrochemical cycle. We first demonstrated the feasibility of electrogenerating the base for our process using analytical electrochemistry, by studying the electrochemical oxidation of benzyl alcohol in the aprotic RTIL 1-octyl-3-methylimidazolium bis­(trifluoromethylsulfonyl)­imide, [C_8_C_1_Im]­[NTf_2_], in the presence of TEMPO and 1-butylimidazole (Figure 1). This RTIL was chosen due to its wide electrochemical window, making it suitable for isolating and analyzing the redox behavior of the other components in the system.^26^ During the positive segment of the first sweep (blue line), a large irreversible oxidation peak appeared at about 1.0 V, which is characteristic of TEMPO-mediated alcohol oxidation in the RTIL, and relatively little reduction current flowed until about −1.2 V, when reduction of the RTIL began.^24,38^ During the second sweep (red line) a large reduction peak appeared at about −0.7 V that was not visible during the first sweep. This peak can be attributed to reduction of [C_4_HIm]^+^ that was formed during the electrooxidation (Step 1 in Scheme 2), liberating the parent C_4_Im and H_2_,^39^ which was then oxidized during the positive sweep at about −0.4 V.^40^ These features demonstrate that the base required for the electrooxidation (C_4_Im) could potentially be generated *in situ*, a strategy that we carried forward in our process development.

**Figure 1:**
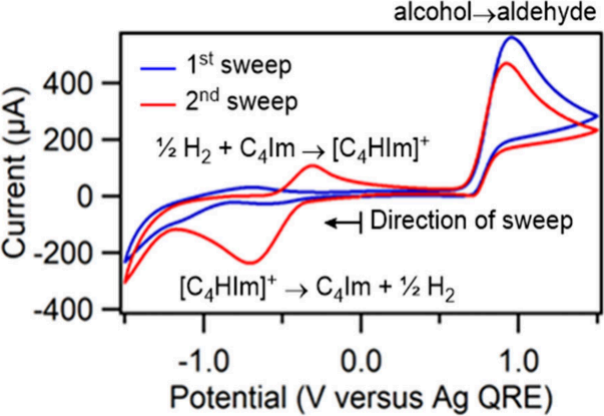
First (blue line) and second (red line) cyclic voltammograms of 0.06 mol dm^–3^ TEMPO, 0.6 mol dm^–3^ benzyl alcohol, and 2.4 mol dm^–3^ 1-butylimidazole dissolved in [C_8_C_1_Im]­[NTf_2_]. The initial potential was 0.0 V. The scan rate was 100 mV s^–1^ and the initial scan direction was negative. The working electrode was a 2 mm diameter Pt disk electrode.

We then tested the suitability of [C_4_HIm]­[(TEMPO)­OSO_3_] for driving electrooxidations using analytical electrochemistry. Figure 2a shows a cyclic voltammogram of [C_4_HIm]­[(TEMPO)­OSO_3_] dissolved in [C_4_HIm]­[NTf_2_] (solid blue line) and that of blank [C_4_HIm]­[NTf_2_] (dashed black line). The anodic peak corresponding to oxidation of [(TEMPO)­OSO_3_]^−^ to [(TEMPO^+^)­OSO_3_]^−^ and the cathodic peak corresponding to [(TEMPO^+^)­OSO_3_]^−^ to [(TEMPO)­OSO_3_]^−^ are labelled a and c, respectively, in Figure 2a. The ratio of the anodic to the cathodic peak current, *i*_p,a_/*i*_p,c_, was close to unity, and *i*_p,a_ and *i*_p,c_ were proportional to the square root of the voltametric scan rate, *ν* ^1/2^, as expected for a freely-diffusing species.^41^ The separation between the anodic and cathodic peak potentials, Δ*E*_p_, was 70 mV, which is slightly higher than expected for an electrochemically-reversible system involving the transfer of a single electron (∼59 mV).^42^ As ohmic-drop compensation was performed using positive-feedback correction during all voltametric measurements, the high Δ*E*_p_ is attributed to sluggish electron transfer across the electrode/RTIL interface, as observed previously during cyclic voltammetry using RTIL electrolytes.^25,38,43^

**Figure 2:**
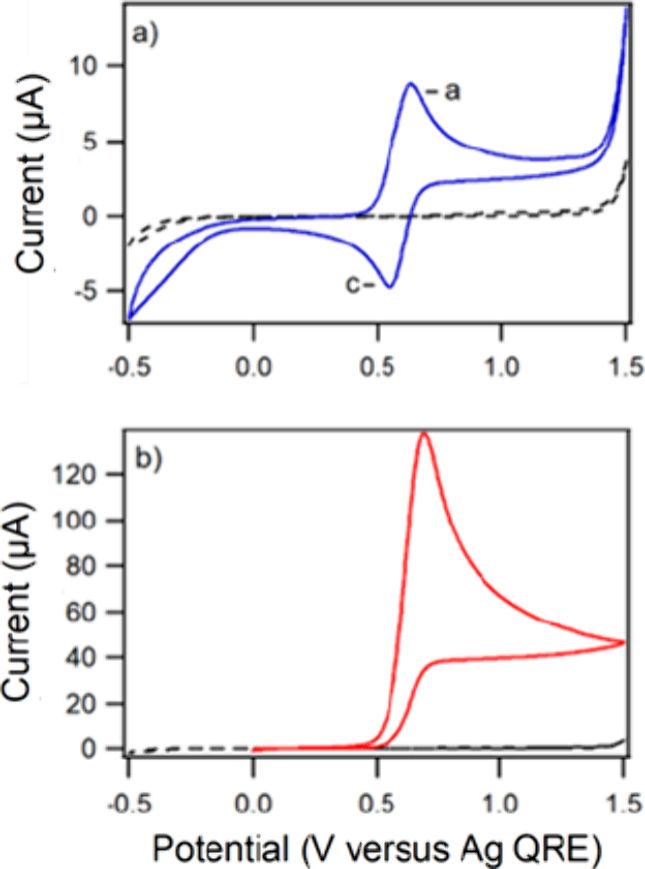
(a) Cyclic voltammograms of 0.06 mol dm^–3^ [C_4_HIm]­[(TEMPO)­OSO_3_ ]­in [C_4_HIm]­[NTf_2_] (solid blue line) and blank [C_4_HIm]­[NTf_2_] (dashed line). (b) Cyclic voltammograms of 0.06 mol dm^–3^ [C_4_HIm]­[(TEMPO)­OSO_3_] and 0.6 mol dm^–3^ benzyl alcohol in [C_4_HIm]­[NTf_2_] (solid red line), and blank [C_4_HIm]­[NTf_2_] (dashed line). The voltammograms were recorded using a 3 mm diameter glassy carbon disk electrode at 5 mV s^–1^ and the initial scan direction was positive in each case.

As shown in Figure 2b, addition of benzyl alcohol to the RTIL electrolyte system caused *i*_p,a_ to increase significantly and *i*_p,c_ to decrease to a negligible level, because of the catalytic reaction between [TEMPO^+^]­[OSO_3_]^−^ and benzyl alcohol, producing benzaldehyde (Step 4 in Scheme 2). The increase in anodic current and disappearance of the reduction current are due to the regeneration of [(TEMPO)­OSO_3_]^−^ during the alcohol oxidation cycle and analogous to that which occurred during the voltammogram in Figure 1. The presence of the reducible [C_4_HIm]^+^ cations within the system, yields the 1-butylimidazole required for the electrooxidation of TEMPO to occur.^37,44,45^ However, in this analytical setup the 1-butylimidazole is generated at the counter electrode, remote from the working electrode, and meaning that its concentration remained relatively low during this analysis. To explore whether our system would benefit from addition of extra 1-butylimidazole, the effects of increasing its concentration were analyzed by performing a kinetic study of the electrooxidation of benzyl alcohol in the [C_4_HIm]­[(TEMPO)­OSO_3_]-[C_4_HIm]­[NTf_2_] system in the presence and absence of 1-butylimidazole. The reaction rate constant, *k*, for the catalytic cycle can be determined using eq 1:^45–47^

1 $$i_{\mathrm{cat}}=nAFC_{T}{\left(D_{A}kC_{A}\right)}^{1/2}$$

where *C*_T_ is the bulk concentration of ([C_4_HIm]­[(TEMPO)­OSO_3_]), *n* is the number of electrons involved in one cycle (2 in this case), *F* is the Faraday constant, *A* the surface area of the working electrode, *D* is the diffusion coefficient of the alcohol, *i*_cat_ is the catalytic current, and *C*_A_ is the concentration of benzyl alcohol. *k* can be derived from the slope of the linear plot of *i*_cat_ *vs*. *C*_A_^1/2^. *D* was determined as 0.317 (± 0.004) × 10^–10^ m^2^ s^–1^ using pulsed field gradient nuclear magnetic resonance spectroscopy (the method for deriving *D* is described in the SI). Figure 3a shows cyclic voltammograms of solutions of different concentrations of benzyl alcohol in [C_4_HIm]­[(TEMPO)­OSO_3_]-[C_4_HIm]­[NTf_2_] without any added 1-butylimidazole. The catalytic current increased linearly as *C*_A_^1/2^ increased, as expected from eq 1 (Figure 3a inset).^45,47^ *k* was 0.052 (± 0.0001) dm^3^ mol^–1^ s^–1^, which is relatively low for TEMPO-mediated alcohol oxidations in RTILs.^26^ By comparison, our previous work yielded *k* for the TEMPO-mediated oxidation of benzyl alcohol in [C_8_C_1_Im]­[NTf_2_] as 0.336 (± 0.004) dm^3^ mol^–1^ s^–1^ and 0.249 (± 0.004) dm^3^ mol^–1^ s^–1^ in acetonitrile containing tetraethylammonium tetrafluoroborate.^26^

**Figure 3:**
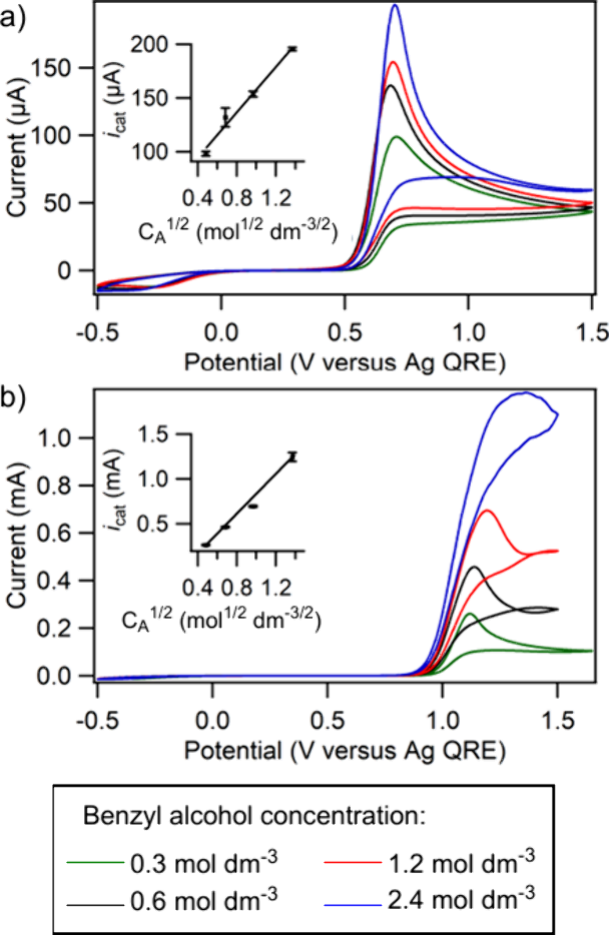
(a) Cyclic voltammograms of 0.06 mol dm^–3^ [C_4_HIm]­[(TEMPO)­OSO_3_] dissolved in [C_4_HIm]­[NTf_2_] containing increasing concentrations of benzyl alcohol (see bottom legend for concentrations). (b) Cyclic voltammograms of 0.06 mol dm^–3^ [C_4_HIm]­[(TEMPO)­OSO_3_] and 2.4 mol dm^–3^ 1-butylimidzole dissolved in [C_4_HIm]­[NTf_2_] containing increasing concentrations of benzyl alcohol. The insets show plots of the catalytic peak current *vs*. the square root of the benzyl alcohol concentration. The voltammograms were recorded using a 3 mm diameter glassy carbon disk electrode at 5 mV s^–1^ and the initial scan direction was positive in each case.

Figure 3b shows the effect of adding 2.4 mol dm^–3^ 1-butylimidazole to benzyl alcohol in [C_4_HIm]­[(TEMPO)­OSO_3_]-[C_4_HIm]­[NTf_2_]. The catalytic currents for each alcohol concentration in Figure 3b are significantly higher than in Figure 3a, due to the higher concentration of base and is consistent with studies involving organic,^38,40^ aqueous^25^ and other RTIL-based media.^24^ *k* increased from 0.052 ± (0.0001) dm^3^ mol^–1^ s^–1^ to 2.37 ± (0.004) dm^3^ mol^–1^ s^–1^ upon addition of 1-butylimidazole (*D* increased to 0.753 (± 0.008) × 10^–10^ m^2^ s^–1^), due to faster deprotonation of the alcohol and hydroxylamine. While addition of extra base to the electrolyte increases the complexity of the [C_4_HIm]­[(TEMPO)­OSO_3_]-[C_4_HIm]­[NTf_2_] system, we describe in the following sections how its use increased yields during electrosyntheses. We note that others have also shown how additives can improve performance during electrooxidations, potentially outweighing the complications associated with additional electrolyte components.^48^ Moreover, we also show that the added 1-butylimidazole can be recovered and reused by converting it to [C_4_HIm]­[NTf_2_].

### Alcohol Electrooxidation in Flow

In the absence of 1-butylimidazole, 32% of benzyl alcohol was converted to benzaldehyde at a flow rate of 0.05 cm^3^ min^–1^ and applied current of 20 mA. Addition of 2.4 mol dm^–3^ 1-butylimidazole more than doubled the yield to 78% under the same conditions. Yields were calculated based on the relative concentrations of benzaldehyde and unreacted benzyl alcohol determined using GC-FID analysis. The scope of the process was then determined by oxidizing a range of primary and secondary alcohols and yields were determined by comparing the relative amount of starting materials and desired products (Scheme 3). In general, benzylic alcohols were oxidized more readily than aliphatic alcohols. More 4-methoxybenzylaldehyde (**2**) than benzylaldehyde (**1**) was converted, as the electron-donating methoxy group increased the hydridic nature of the benzylic position, favoring the rate limiting H-transfer within the alkoxide-TEMPO^+^ adduct.^45,48–50^ Electron-poor benzylic alcohols, such as 4-nitrobenzyl alcohol and 4-bromobenzyl alcohol, gave lower yields of 4-nitrobenzaldehyde (**3**) and 4-bromobenzaldehyde (**4**), respectively. A Hammett plot (Figure 4) for *para*-substituted benzylic alcohols showed a linear relationship between the Hammet substituent and conversion yield.^51^ Notably, the product mixture obtained after oxidation of 4-nitrobenzylalcohol was dark brown in color (in contrast to the light orange-colored starting reactant mixture). Brown and co-workers have previously reported a particularly low yield of 4-nitrobenzylalcohol when conducting the TEMPO-mediated alcohol oxidation electrolysis in the undivided Ammonite8 flow electrolysis cell. It was believed that the low yield could be a result of the nitro group potentially reacting at the negative electrode as a side reaction.^25^

**Scheme 3:**
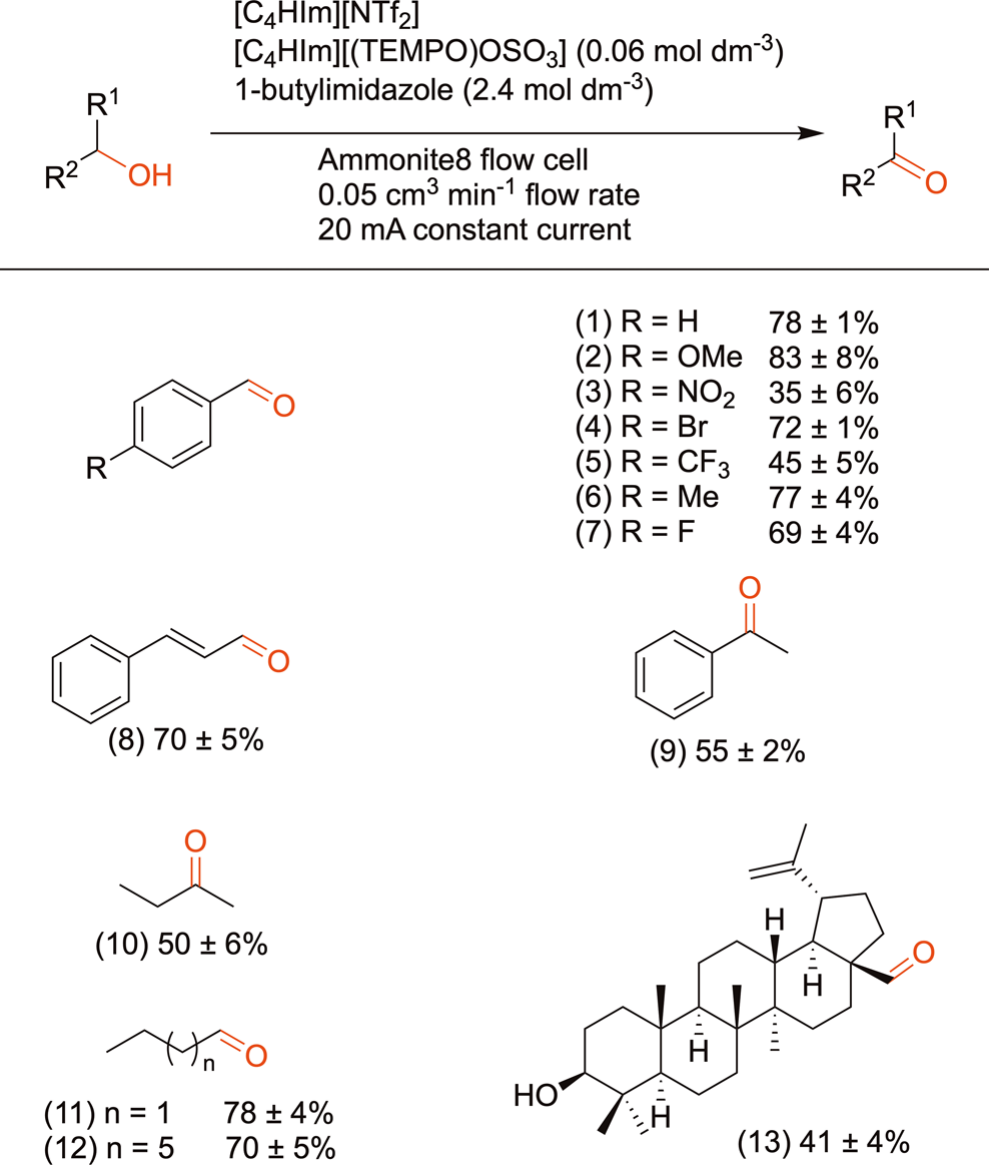
Products Formed during TEMPO-RTIL-Mediated Electrooxidation of 0.2 mol dm^–3^ of Various Alcohols under Flow^a^

a Percentage yields were obtained from NMR spectra, comparing the relative concentrations of the starting materials and respective products. Electrolysis of each alcohol was conducted in triplicate, and average yields are shown.

**Figure 4:**
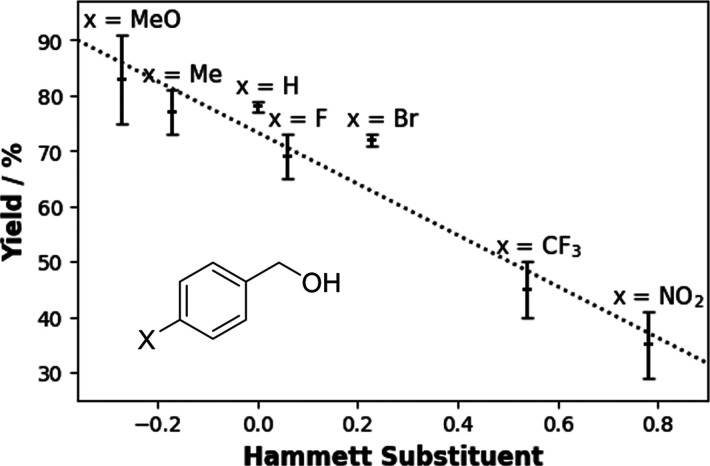
Hammett plot for the electrooxidation of 4-substituted benzyl alcohols.

Under alkaline conditions, TEMPO exhibits strong chemoselectivity towards the oxidation of primary alcohols over secondary alcohols.^17^ This selectivity has been attributed to steric effects in the formation of the alkoxide-TEMPO^+^ adduct, the stability of which decreases as crowding increases.^45^ These effects were mirrored in the lower yields of products (**9**) and (**10**) than (**1**) and (**11**), respectively, demonstrating that the [C_4_HIm]­[NTf_2_]-[C_4_HIm]­[(TEMPO)­OSO_3_] system is also selective towards oxidation of primary alcohols. As the length of the alkyl chains of the substrates increased, forming the carbonyl products butanal (**11**) and octanal (**12**), yields decreased. This decreased yield has previously been observed and is also attributed to the effects of steric hindrance as the chain length increases.^25^

Betulin is a triterpene containing primary and secondary alcohol groups and was studied to further probe the reaction selectivity. The oxidation of betulin is also of interest as it is a precursor to betulinic acid, which has shown anti-tumour activity against melanoma and anti-HIV activity in phocytic cells.^52,53^ 41% of the betulin was converted to betulin-28-aldehyde (**13**). However, it should be noted that betulin did not fully dissolve in the mixture of [C_4_HIm]­[(TEMPO)­OSO_3_]-[C_4_HIm]­[NTf_2_] and 1-butylimidazole. Considering the design space of RTILs, there are future prospects for designing an RTIL that can fully dissolve betulin.^54^

### Recyclability of [C_4_HIm]­[(TEMPO)­OSO_3_] and [C_4_HIm]­[(TEMPO)­OSO_3_]

Benzaldehyde, 1-butylimidazole and unreacted benzyl alcohol were extracted from the [C_4_HIm]­[(TEMPO)­OSO_3_]-[C_4_HIm]­[NTf_2_] system by distillation *in vacuo*, which did not require the use of any volatile organic solvents or washing of the RTIL phase. The [C_4_HIm]­[(TEMPO)­OSO_3_]-[C_4_HIm]­[NTf_2_] system was then recharged with fresh substrate and the electrolysis was repeated 10 times. After the 10^th^ recycling of the [C_4_HIm]­[(TEMPO)­OSO_3_]-[C_4_HIm]­[NTf_2_] system, 92% of the benzyl alcohol was converted to benzaldehyde (Figure 5). This demonstrates that the [C_4_HIm]­[(TEMPO)­OSO_3_]-[C_4_HIm]­[NTf_2_] system could be recycled and reused. [C_4_HIm]­[NTf_2_] could be regenerated from extracted 1-butylimidazole by reaction with HCl to form [C_4_HIm]­Cl, followed by metathesis with Li­[NTf_2_].

**Figure 5:**
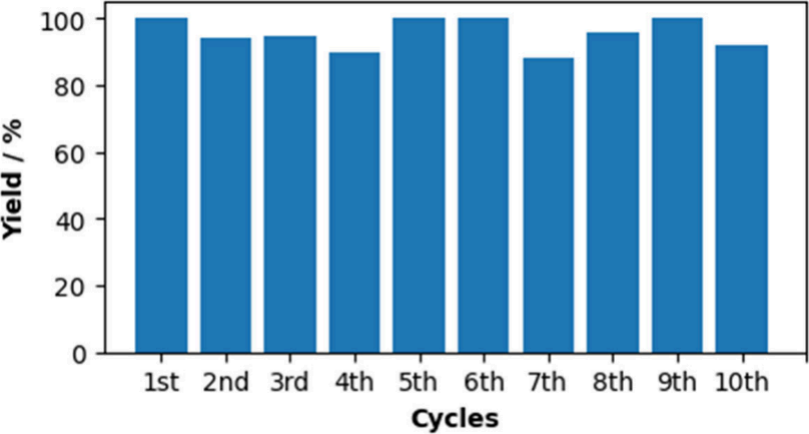
Percentage yield of benzaldehyde from benzyl alcohol using the [C_4_HIm]­[(TEMPO)­OSO_3_]-[C_4_HIm]­[NTf_2_] system after each of 10 recycles of the RTIL.

[C_4_HIm]­[NTf_2_] and [C_4_HIm]­[(TEMPO)­OSO_3_] are immiscible with commonly used non-polar organic solvents such as toluene and hexane, so an alternative procedure to distillation in vacuo is solvent extraction of the carbonyl products. We examined the feasibility of this approach by using hexane to coextract octanal (**12**) and 1-butylimidazole from the [C_4_HIm]­[NTf_2_]-[C_4_HIm]­[(TEMPO)­OSO_3_] system. Subsequent aqueous extraction of 1-butylimidazole (as [C_4_HIm]­Cl) from the hexane by addition of HCl allowed octanal (**12**) and unreacted alcohol to be recovered from the hexane extract under reduced pressure.

## Conclusions

A recyclable ionic liquid-based mediator/solvent system has been developed for electrochemical alcohol oxidations in an electrochemical flow reactor. The anionic component of a TEMPO-grafted room temperature ionic liquid catalyzed oxidation of the alcohols at the positive electrode of the reactor. This electrooxidation is paired with the electroreduction of the cationic imidazolium-based components of the ionic liquid to generate some of the base required during the reaction. The use of such multi-functional room temperature ionic liquid systems as electrochemical mediator/solvents could help mitigate the major sources of waste typically generated in TEMPO-mediated alcohol electrooxidation, including the organic solvent (if distillation is used for the work-up), electrolyte, catalysts, and hazardous by-products. Considering the vast number of room temperature ionic liquids potentially available, the prospects for developing task-specific ionic liquids suited to other electrosynthetic processes are exciting and could contribute to the development of sustainable electrosynthetic processes, as well as unlock potentially new and innovative synthetic pathways.

## Supplementary Material





## Notes

NMR yields, materials & methods, syntheses, and additional experimental and characterization data (PDF)
Crystallographic information file (CIF) for the RTIL-supported TEMPO derivative, including experimental parameters and data (CIF)
