# Supplementary material for: Recyclable Multifunctional Ionic Liquids for Sustainable Electroorganic Oxidations
Source: ACS Electrochem. 2025 Jul 9;1(9):1659–65. doi: 10.1021/acselectrochem.5c00108 (PMC12415826; doi:10.1021/acselectrochem.5c00108)
Supplement: Supplementary file 1 [file ec5c00108_si_001.pdf]

Supporting Information

Recyclable Multifunctional Ionic Liquids for Sustainable Electroorganic Oxidations

Astrid E. Delorme,[1,2] Andrew Jordan,[2] Jessica Streets,[2] Victor Sans,[3,4] Stephen P. Argent,[1] Helen F. Sneddon,[5] Peter Licence*[1,2] and Darren A. Walsh*[1,2]

[1] School of Chemistry, University of Nottingham, University Park, Nottingham NG7 2RD, UK.

[2] GSK Carbon Neutral Laboratory for Sustainable Chemistry, University of Nottingham, Jubilee Campus, Nottingham NG7 2TU, UK

[3] Faculty of Engineering, University of Nottingham, University Park, Nottingham NG7 2RD, UK

[4] Institute of Advanced Materials (INAM), Universitat Jaume I, Avda. Sos Bainat s/n, 12071, Castellon, Spain

[5] Department of Chemistry, University of York, Heslington, York, YO10 5DD, UK

*email: darren.walsh@nottingham.ac.uk; Tel: 0115 846 7495

*email: peter.licence@nottingham.ac.uk; Tel. 0115 846 6176

Table of Contents

**Page S2**	Reagents and Chemicals
**Page S2**	Nuclear Magnetic Resonance Spectroscopy
**Page S2**	Gas Chromatography
**Page S3**	Single Crystal X-Ray Diffraction
**Page S4**	Electrochemical Methods
**Page S4**	Synthesis of Sodium (2,2,6,6-tetramethylpiperidin-N-oxyl)-4-sulfate, Na[(TEMPO)OSO_3]
**Page S4**	Synthesis of 1-butylimidazolium chloride, [C_4HIm]Cl
**Page S5**	Synthesis of 1-butylimidazolium bis(trifluoromethanesulfonyl)imide, [C_4HIm][NTf_2]
**Page S6**	Synthesis of 1-butylimidazolium (2,2,6,6-tetramethylpiperidin-N-oxyl)-4-sulfate, [C_4HIm][(TEMPO)OSO_3]
**Page S6**	Crystallographic Data for AJGSKA $C_{16}H_{30}N_3O_5S$
**Page S8**	Nuclear Magnetic Resonance Spectra
**Page S15**	References

Reagents and Chemicals. TEMPO (Sigma Aldrich, 98%), TEMPOL (Sigma Aldrich, 97%), HCl (Fisher Scientific, 37% in water), sulfuric acid (Sigma Aldrich, 98%), 1-butylimidazole (Sigma Aldrich, 98%), benzyl alcohol (Sigma Aldrich, 99.8%), 1-octanol (Acros Organics, 99%), 2-phenylethanol (Sigma Aldrich, 99%), 2-butanol (Sigma Aldrich, 99%), 4-nitrobenzyl alcohol (Acros Organics, 99%), 4-methoxybenzyl alcohol (Sigma Aldrich, 98%), 4-bromobenzyl alcohol (Sigma Aldrich, 99%), cinnamyl alcohol (Sigma Aldrich, 98%), 1-butanol (Sigma Aldrich, ≥ 99.5%), and betulin (Sigma Aldrich, ≥ 98%) were used as received.

Na[TEMPOOSO$_3$] was prepared according to literature procedure.[1] Room temperature ionic liquids were fully characterized by ^{1}H, ^{13}C and ^{19}F NMR spectroscopy and ESI-MS (Bruker MicroTOF spectrometer). All ionic liquids were dried *in vacuo* (p~10^{-4}–10^{-6} mbar) and then stored under Ar. The water contents of the ionic liquids were measured prior to electrochemical measurements using Karl-Fisher analysis and were <200 ppm. No halide impurities were detected using anion chromatography (DIONEX ICS-5000).

Nuclear Magnetic Resonance Spectroscopy. NMR spectroscopy was carried out using a Bruker DPX-400 spectrometer. Reaction conversions were calculated using the ratio of the alcohol and aldehyde/ketone peaks. An exemplar reaction mixture NMR spectrum of each alcohol is included in Pages S13-S19. PFG-NMR measurements were performed using the pulse program ledbpgp2s (bipolar dosy experiment) was used with Δ = 149.9000 m and δ = 5.000 m, and experiments were run with 32 points and 16 scans for each. A solution of 0.6 mol dm^{-3} benzyl alcohol and 2.4 mol dm^{-3} 1-butylimidazole in ionic liquid were added to the coaxial insert which was then added into the NMR tube with deuterated acetone. Measurements were performed in triplicate.

Gas Chromatography. Quantification of products were carried out on a TRACE^{TM} 1310 Thermo Scientific^{TM} gas chromatograph (GC) equipped with a flame ionization detector (FID). A Thermo Scientific^{TM} TraceGOLD TG-17MS GC column with an inner diameter of 0.25 mm and a length of 30 m was used. The product formation and reagent consumption were determined by collecting 50 µL of the product mixture, extracting the alcohol, aldehyde/ketone and tert-butylbenzene, into 5 cm^3 of toluene. The initial oven temperature was 80 °C for 2 min and was ramped to 120 °C at 10 °C min^{-1} and then kept at 180 °C for 5 min A solvent delay of 4 min was used. Calibration standard solutions were prepared containing known amounts of tert-butylbenzene and varying concentrations of benzyl alcohol and benzaldehyde and are presented in Figure S1.

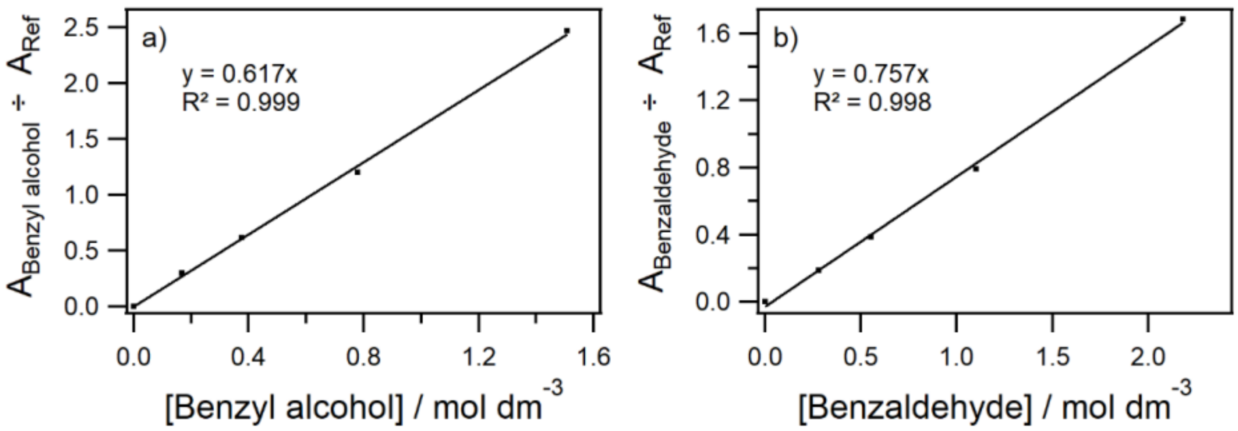

Figure S1. GC-FID Calibration curves for a) benzyl alcohol, and b) benzaldehyde. Calibration curves were obtained by measuring the ratio of the peak area for benzyl alcohol or benzaldehyde at each concentration to that of the reference. Tert-butylbenzene was used as the reference.

Single Crystal X-Ray Diffraction. A single crystal was selected and mounted using Fomblin® (YR-1800 perfluoropolyether oil) on a polymer-tipped MiTeGen MicroMountTM and cooled rapidly to 120 K in a stream of cold N_2 using an Oxford Cryosystems open flow cryostat.[2] Single crystal X-ray diffraction data were collected on an Oxford Diffraction SuperNova Duo diffractometer (Atlas CCD area detector, mirror-monochromated Mo-K_α radiation source; λ = 0.71073 Å; ω scans). Cell parameters were refined from the observed positions of all strong reflections and absorption corrections were applied using a Gaussian numerical method with beam

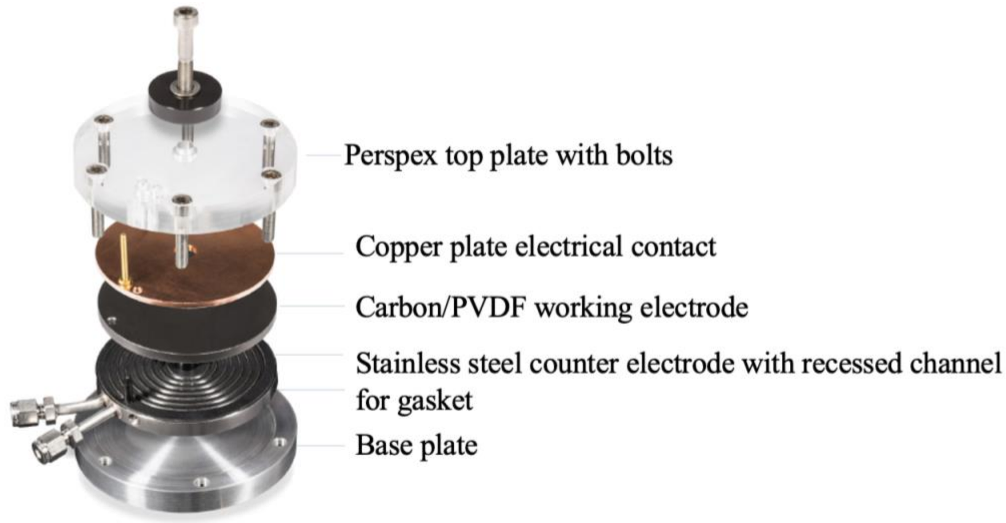

Figure S2. Ammonite8 electrochemical cell used for flow electrolysis. Electrode surface area of 20 cm^2, microfluidic channel volume of 1 cm^3.[6]

profile correction (CrysAlisPro).[3] The structure was solved within Olex2[4] by dual-space iterative methods (SHELXT).[5] The final structure was checked with checkCIF (https://checkcif.iucr.org). CCDC-2130483 contains the supplementary data for these compounds. These data can be obtained free of charge from The Cambridge Crystallographic Data Centre via www.ccdc.cam.ac.uk/data_request/cif.

Electrochemical methods. A schematic diagram of the Ammonite8 flow cell used for flow electrosynthesis is shown in Figure S2.

Synthesis of Sodium (2,2,6,6-tetramethylpiperidin-N-oxyl)-4-sulfate, Na[(TEMPO)OSO₃].[1] 5.0 g of TEMPOL was ground to a fine powder and was then added in small portions to 10 cm^3 of concentrated (98%) H_2SO_4. The solution was stirred for 20 min and then added dropwise to a solution of aqueous $NaHCO_3$ (47.3 g in 500 cm^3 water). The aqueous phase was washed twice with ethyl acetate (150 cm^3) to remove any unreacted TEMPOL and/or the hydrolysed reaction product. Water was removed under reduced pressure. Acetone (150 cm^3) was added to the lightly yellow coloured solid and the resulting dispersion was stirred until the solid was colourless (approximately 1 h). Acetone was removed from the filtrate under reduced pressure, to yield a dark-red waxy solid (6.5 g, 82%).

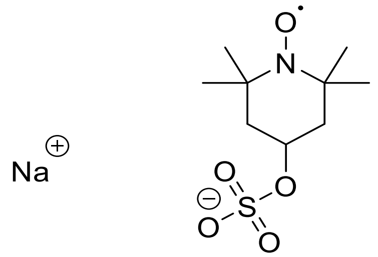

ESI-MS (-ve) $[C_9H_{17}NO_5S]^-$: calcd 251.0833 m/z found 251.0835 m/z.

CHN Analysis: calcd. for $Na[(TEMPO)OSO_3]$, ($NaC_9H_{17}NO_5S$): C 37.22%, H 5.90%, N 4.82% found: C 36.90%, H 6.60%, N 4.69%.
Data in agreement with literature.[1]

Synthesis of 1-butylimidazolium chloride, [C₄HIm]Cl.[7] 200 cm^3 of a 1.0 mol dm^{-3} aqueous HCl was added dropwise to 200 cm^3 of 1 mol dm^{-3} aqueous 1-butylimidazole. The temperature of the solution was kept below 20 °C during addition. The mixture was then stirred at room temperature for 30 min. The water was removed under reduced pressure and the product was dried *in vacuo* at 40 °C for 12 h to yield a white crystalline solid product (31 g, 97%).

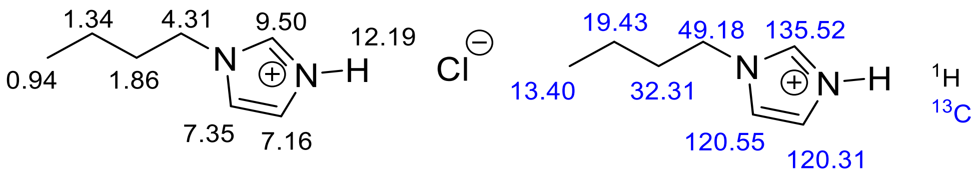

1H NMR (400 MHz, $CDCl_3$) δ ppm: 12.19 (br. s, 1 H), 9.50 (m, 1 H), 7.35 (t, J = 1.8 Hz, 1 H), 7.16 (t, J = 1.8 Hz, 1 H), 4.31 (t, J = 7.3 Hz, 2 H), 1.86 (m, 2 H), 1.34 (m, 2 H), 0.94 (t, J = 7.4 Hz, 3 H).

13C NMR (101 MHz, CDCl3) δ ppm: 135.52, 120.55, 120.31, 49.18, 32.31, 19.43, 13.40.

ESI-MS (+ve) $[C_7H_{13}N_2]^+$: calcd 125.1073 m/z found 125.1074 m/z.

Data in agreement with literature.[7]

Synthesis of 1-butylimidazolium bis(trifluoromethanesulfonyl)imide, [C4HIm][NTf2].[7] [C4HIm]Cl (16.0 g, 0.1 mol) was dissolved in water (150 cm^3) and stirred. A solution of Li[NTf2] (34.5 g, 0.12 mol) and water (150 cm^3) was added to the solution. The solution was stirred overnight at room temperature. Dichloromethane (200 cm^3) was added to the mixture and the organic layer was washed with water (4 × 50 cm^3). The organic layer was separated and the solvent was removed under reduced pressure. The crude liquid was dried *in vacuo* at 40 °C for 12 hr to yield a clear viscous liquid (39.6 g, 98%).

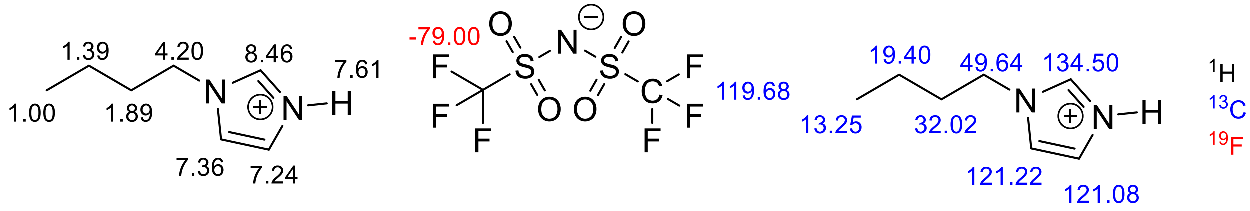

1H NMR (400 MHz, $CDCl_3$) δ ppm: 8.46 (m, 1 H), 7.61 (br. s, 1 H), 7.36 (t, J = 1.8 Hz, 1 H), 7.24 (t, J = 1.8 Hz, 1 H), 4.20 (t, J = 7.4 Hz, 1 H), 1.89 (m, 2 H), 1.39 (m, 2 H), 1.00 (t, J = 7.4 Hz, 3 H).

13C NMR (101 MHz, CDCl3) δ ppm: 134.50, 121.22, 121.08, 119.68 (q, J = 322 Hz), 49.64, 32.02, 19.40, 13.25.

19F NMR (282 MHz, $CDCl_3$) δ ppm: -79.00 (s, 6 F).

ESI-MS (+ve) $[C_7H_{13}N_2]^+$: calcd 125.1073 m/z found 125.1075 m/z.

ESI-MS (-ve) $[C_9H_{17}NO_5S]^-$: calcd 279.9178 m/z found 279.9184 m/z.

Data in agreement with literature.[7]

Synthesis of 1-butylimidazolium (2,2,6,6-tetramethylpiperidin-N-oxyl)-4-sulfate, [C₄HIm][(TEMPO)OSO₃]. Na[(TEMPO)OSO_3] (6.5 g, 0.02 mol) was dissolved in water (50 cm^3) and stirred. The solution was stirred and a solution of [C_4HIm]Cl (3.2 g, 0.02 mol) and ultrapure water (50 cm^3) was added. The solution was stirred overnight at room temperature. 1-butanol was (100 cm^3) was added to the mixture and washed once with ultrapure water (50 cm^3). The organic layer was separated and the solvent was removed under reduced pressure. The crude solid was dried *in vacuo* at 40 °C for 12 hr to yield an orange waxy solid (5.0 g, 67%).

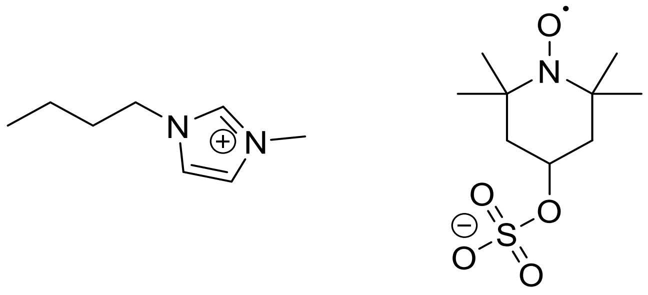

ESI-MS (+ve) $[C_7H_{13}N_2]^+$: calcd 125.1073 m/z found 125.1080 m/z.

ESI-MS (-ve) $[C_9H_{17}NO_5S]^-$: 251.0833 m/z found 251.0833 m/z.

CHN Analysis: calcd. for [C_4HIm][(TEMPO)OSO_3] ($C_{16}H_{30}N_3O_5S$): C 51.00%, H 8.05%, N 11.16% found: C 51.02%, H 8.17%, N 11.09%.

IR ν_{max} cm^{-1}: 2934.27, 1462.39, 1250.80, 1219.04, 1056.63, 964.46, 914.70, 861.42, 791.92, 628.80, 598.36, 561.91.

Crystallographic Data for AJGSKA $C_{16}H_{30}N_3O_5S$ (Figure S3). (M =376.49 g/mol); monoclinic, space group P2_1/n (no. 14), a = 6.0279(4) Å, b = 21.9811(12) Å, c = 15.1206(11) Å, β = 95.962(7)°, V = 1992.6(2) Å^3, Z = 4, T = 120(2) K, μ(Mo Kα) = 0.192 mm^{-1}, $Dcalc$ = 1.255 g/cm^3, 14129 reflections measured (7.044° ≤ 2Θ ≤ 56.932°), 4351 unique (R_{int} = 0.0281, R_{sigma} = 0.0309) which were used in all calculations. The final R_1 was 0.0393 (I > 2σ(I)) and wR_2 was 0.1020 (all data). The imidazolium hydrogen atom H19 was refined with the N-H distance restrained to a target value of 0.88 Å (DFIX, esd 0.02 Å). All other hydrogen atoms in the structure were geometrically placed and refined with a riding model.

Three-fold conformational disorder was modelled in the imidazolium butyl moiety. The occupancies of the three disorder components were refined and restrained to sum to unity using a linear restraint (SUMP, esd 0.01 Å) resulting in values of 0.55(1), 0.24(1) and 0.21(1). The C-C bonds of the disordered butyl chains were all restrained to have target values of 1.5 Å (DFIX, esd 0.02 Å). The N-C bonds of the butyl disorder components were all restrained to have similar lengths (SADI). Rigid bond and similarity restraints were applied to the anisotropic displacement parameters of all the disordered atoms in the structure (RIGU, SIMU). The anisotropic displacement parameters of the three N-pendant carbon atoms were constrained to be identical (EADP). The anisotropic displacement parameters of butyl carbon atoms C34A, C33B and C34B were restrained to have more isotropic character (ISOR).

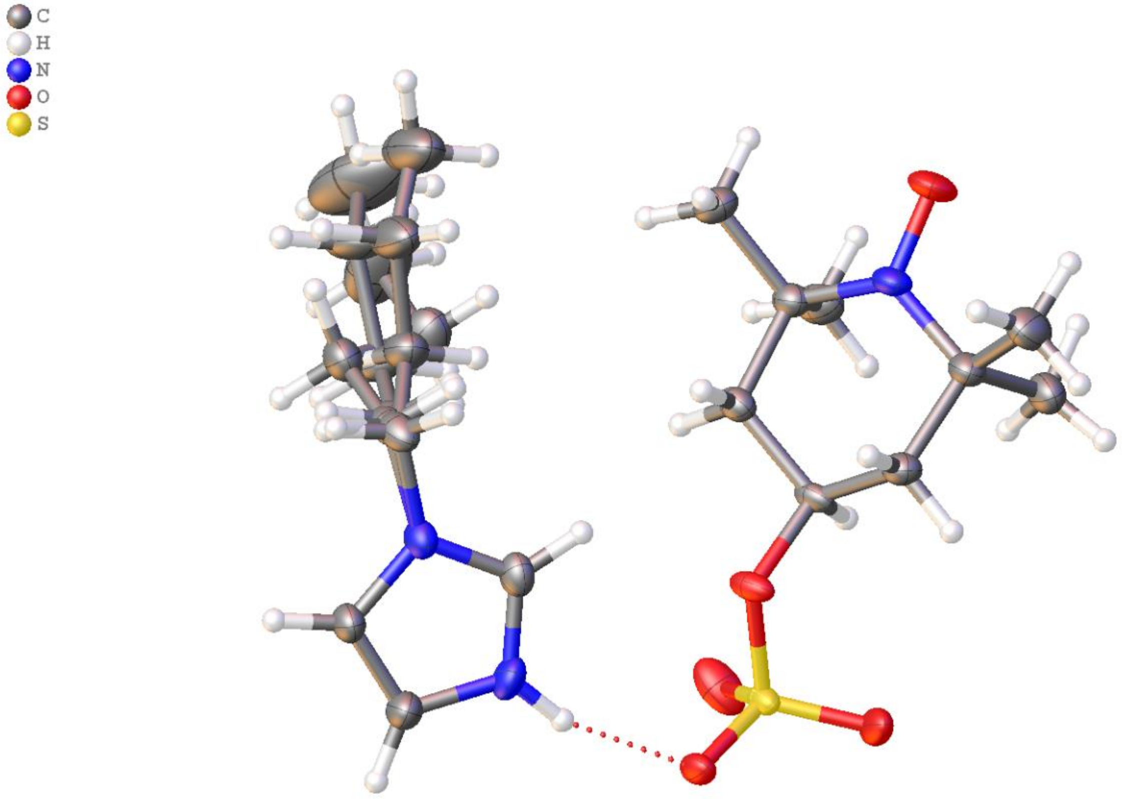

Figure S3. Crystal structure of [C_4HIm][(TEMPO)OSO_3] showing hydrogen bonding as the red dashed line. CCDC deposition number 2130483.

^{1}H NMR Spectrum of Benzyl alcohol, 1

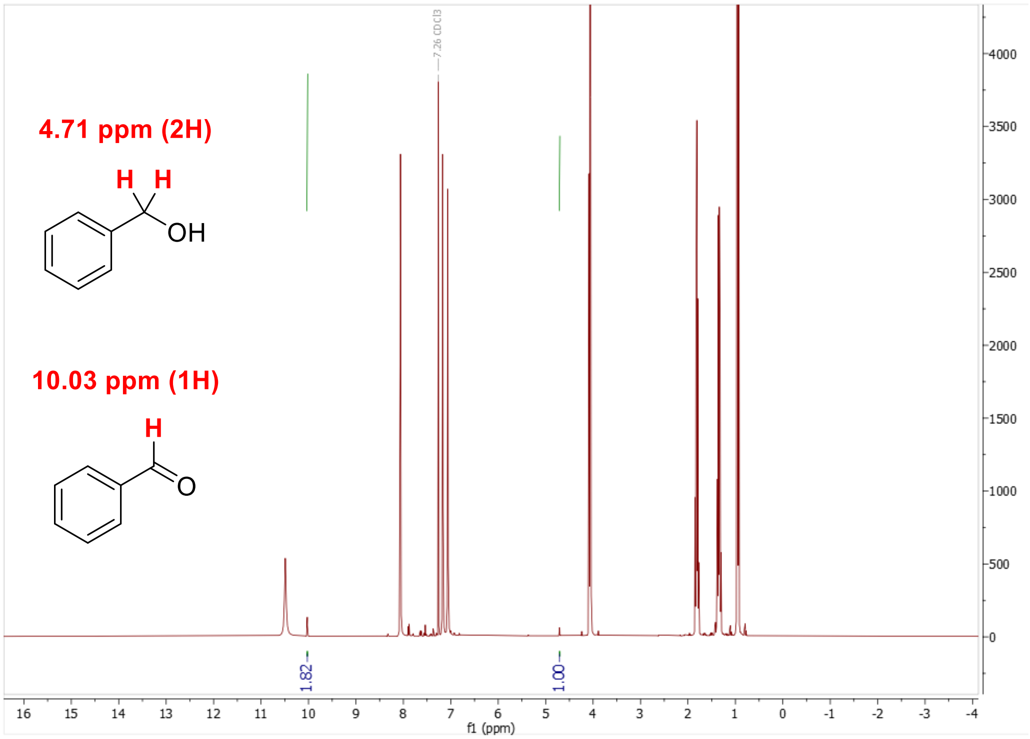

^{1}H NMR spectrumof (4-methoxyphenyl)methanol, 2

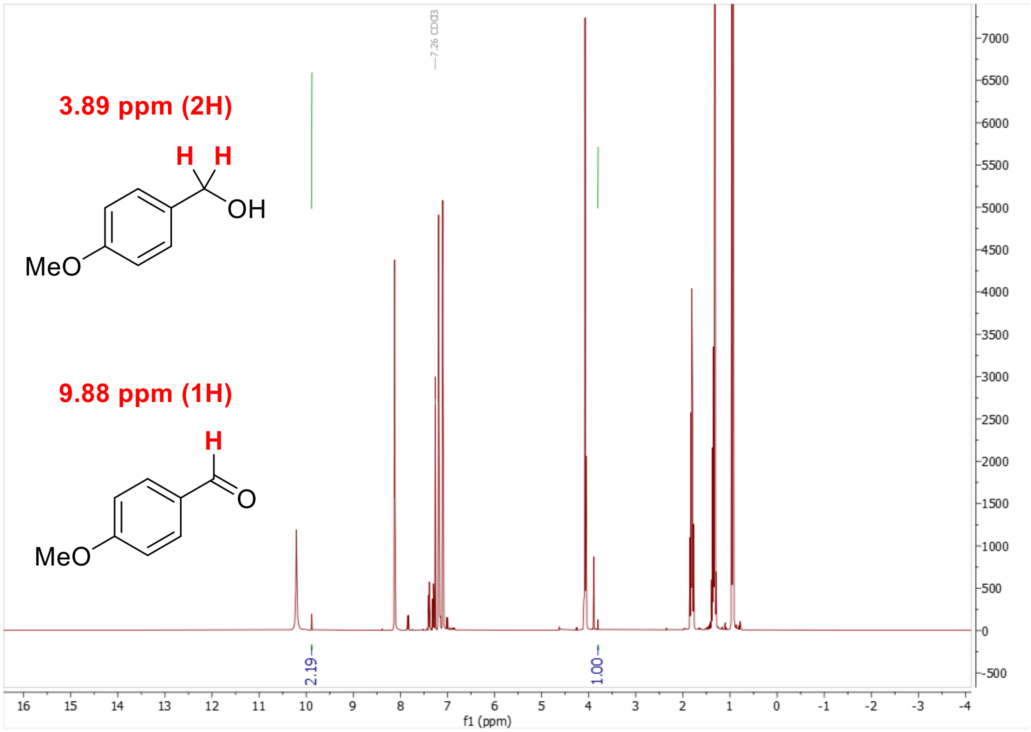

^{1}H NMR spectrum of (4-nitrophenyl)methanol, 3

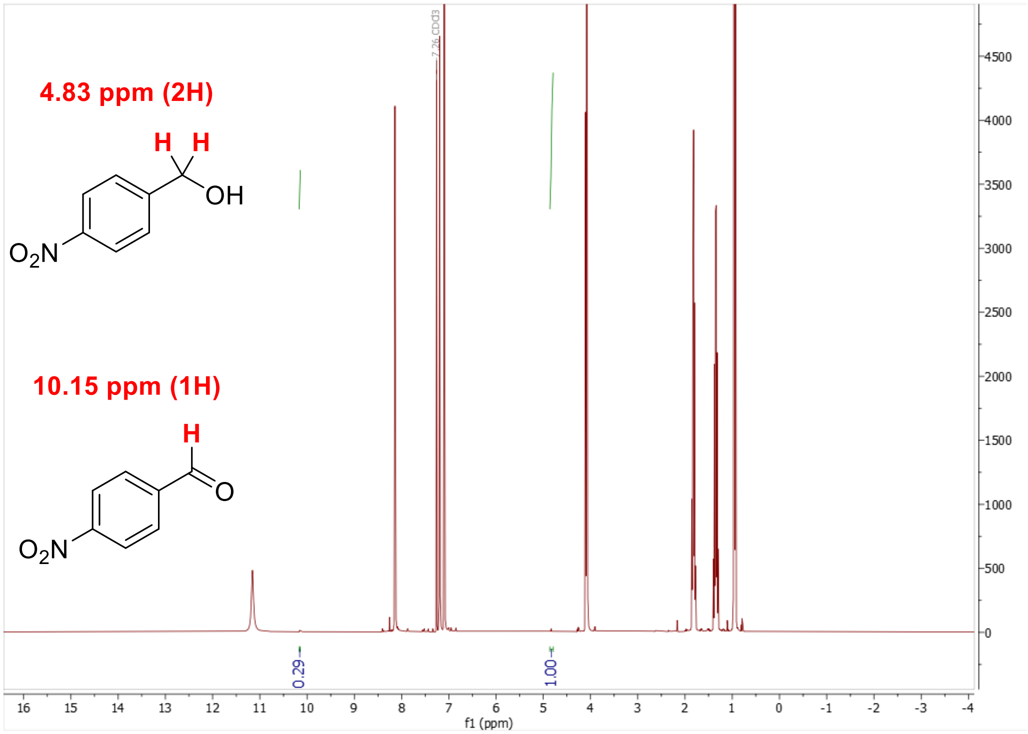

^{1}H NMR sprectrum of (4-bromophenyl)methanol, 4

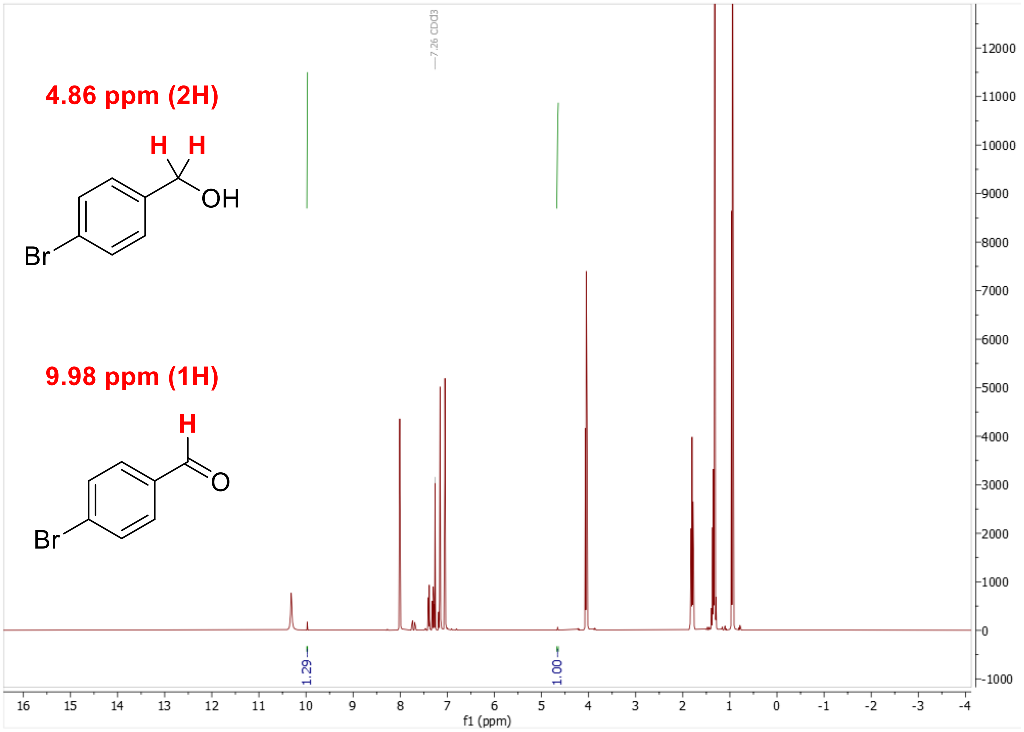

^{1}H NMR spectrum of (4-(trifluoromethyl)phenyl)methanol, 5

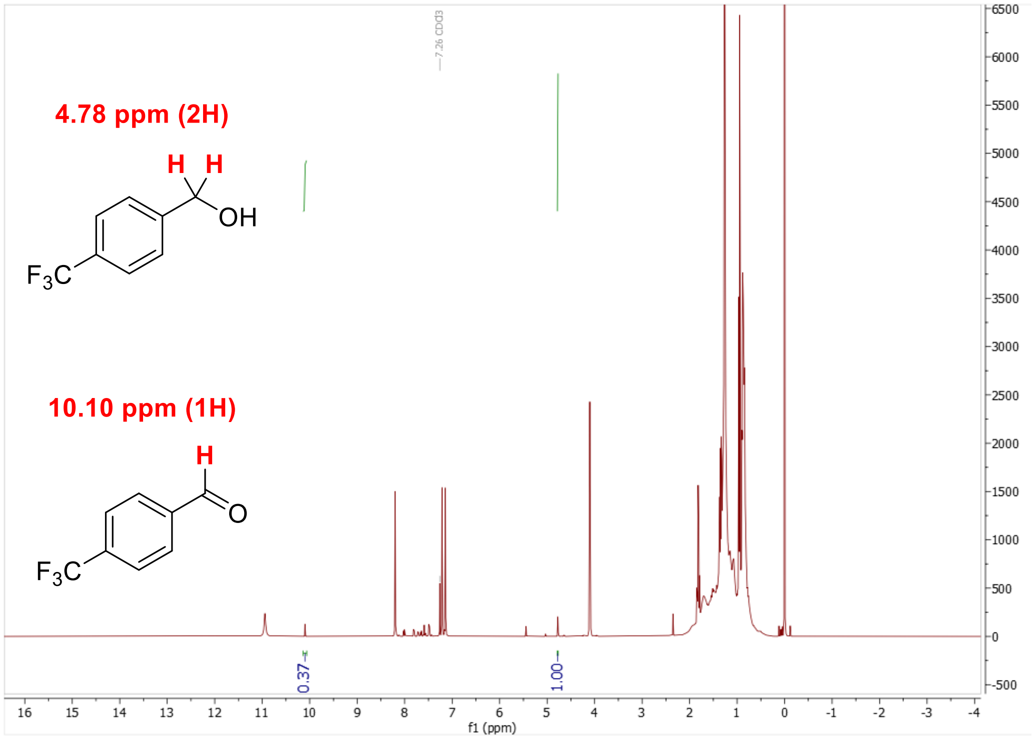

^{1}H NMR spectrum of p-tolylmethanol, 6

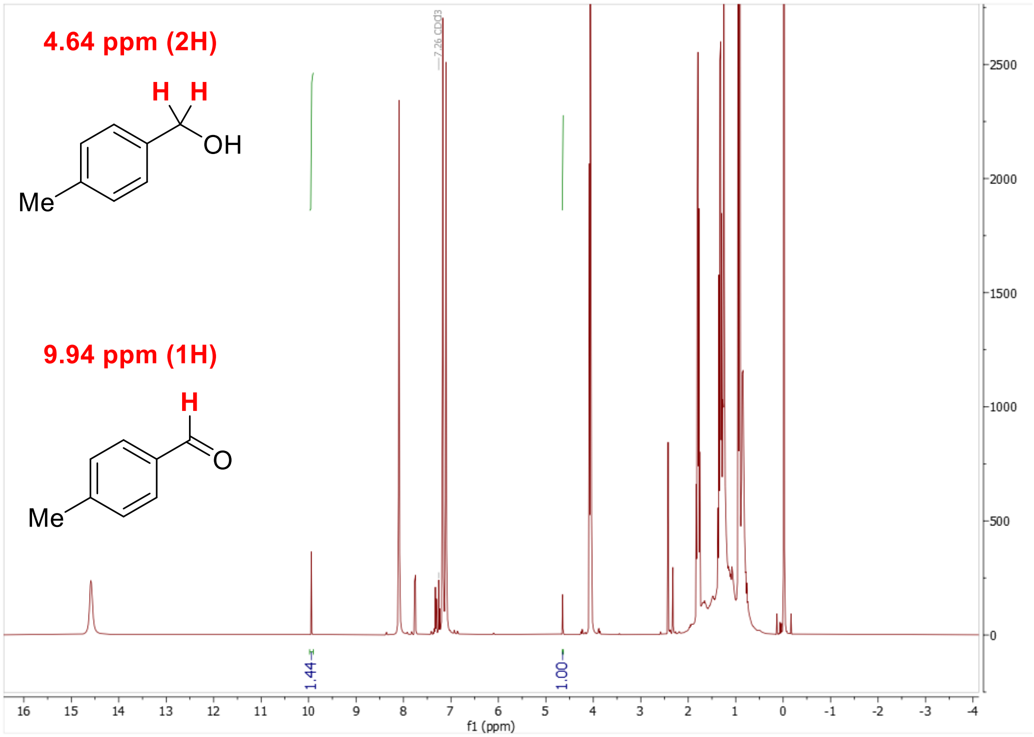

^{1}H NMR spectrum of (4-fluorophenyl)methanol, 7

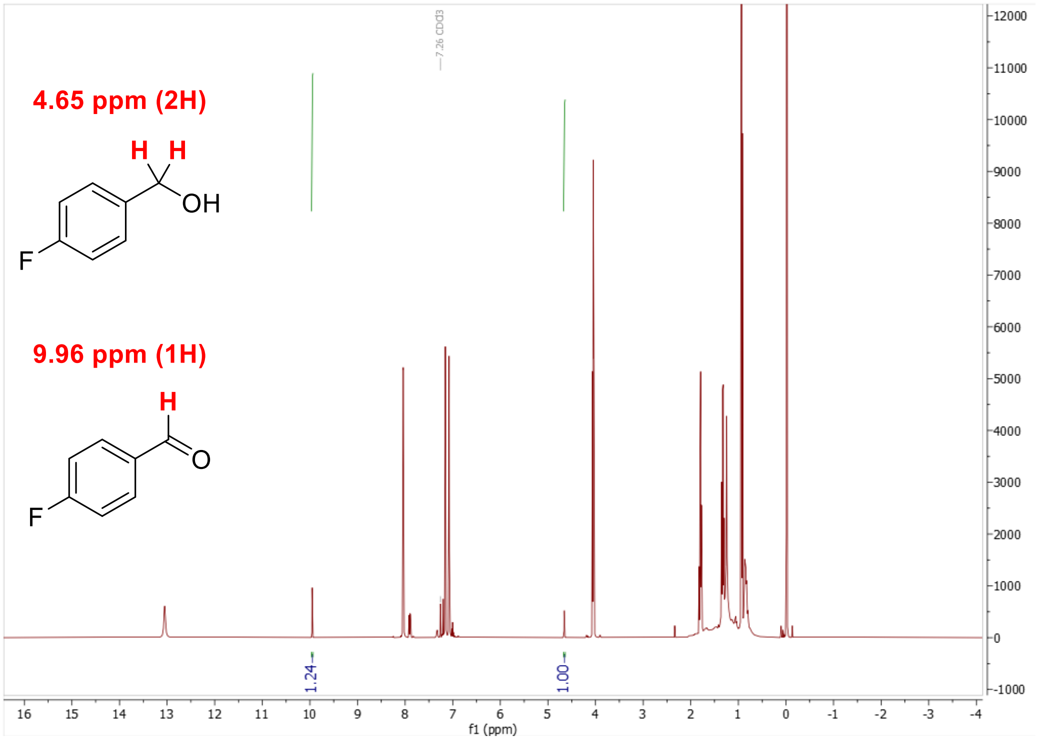

^{1}H NMR spectrum of cinammyl alcohol, 8

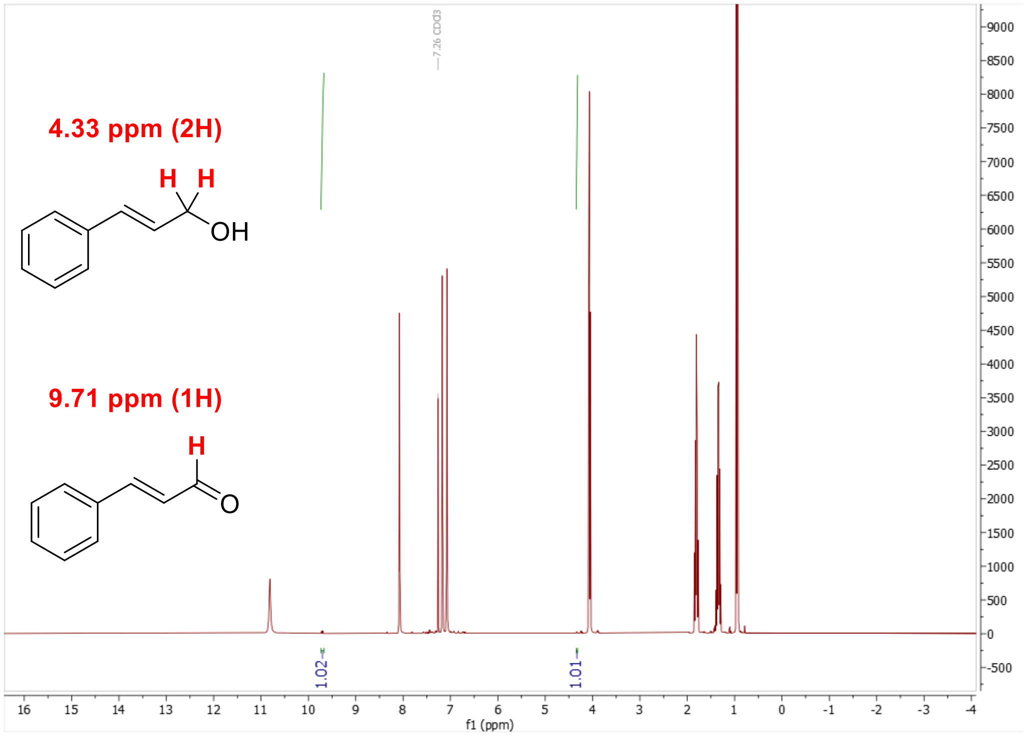

^{1}H NMR spectrum of 1-phenylethan-1-ol, 9

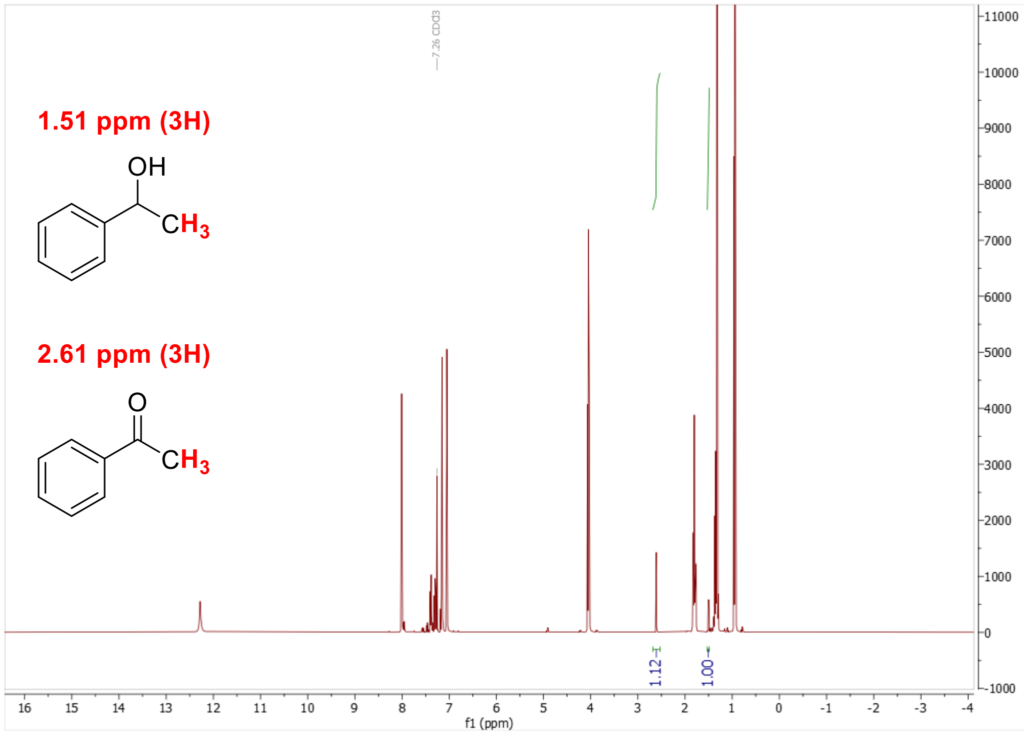

^{1}H NMR spectrum of buntan-2-ol, 10

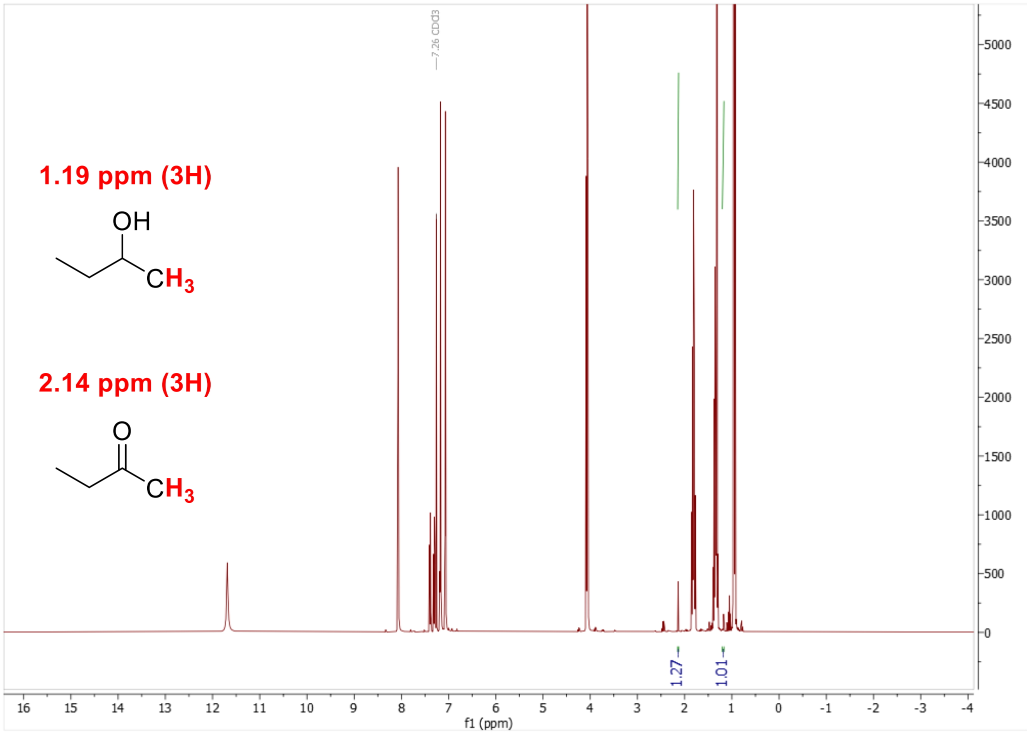

^{1}H NMR spectrum of butan-1-ol, 11

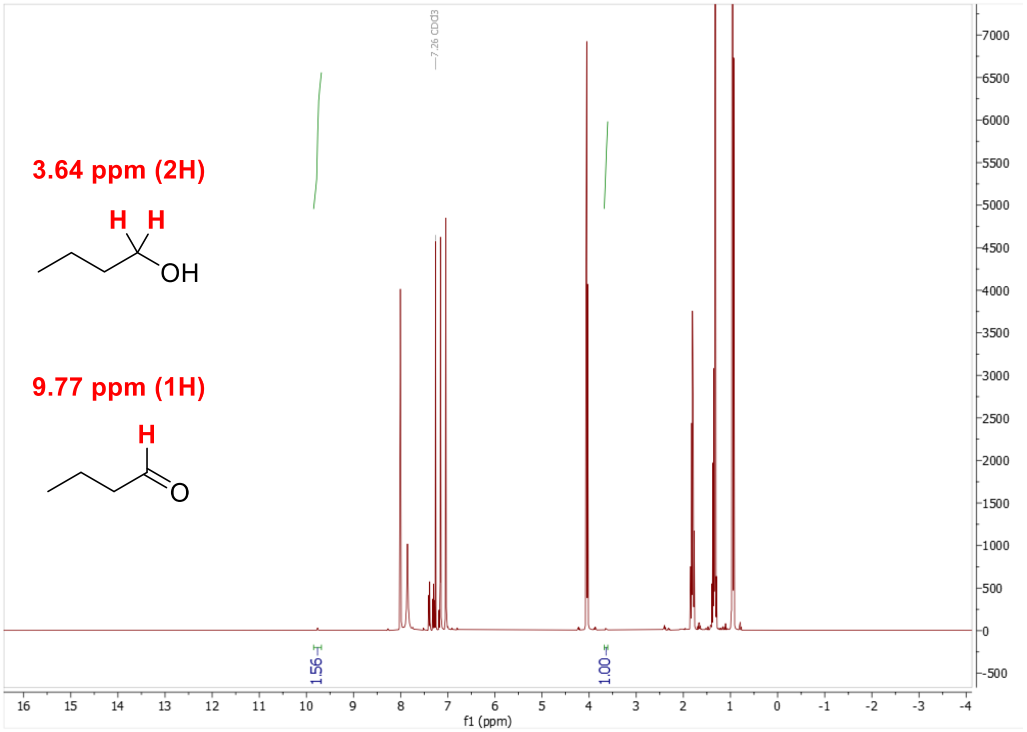

^{1}H NMR spectrum of octan-1-ol, 12

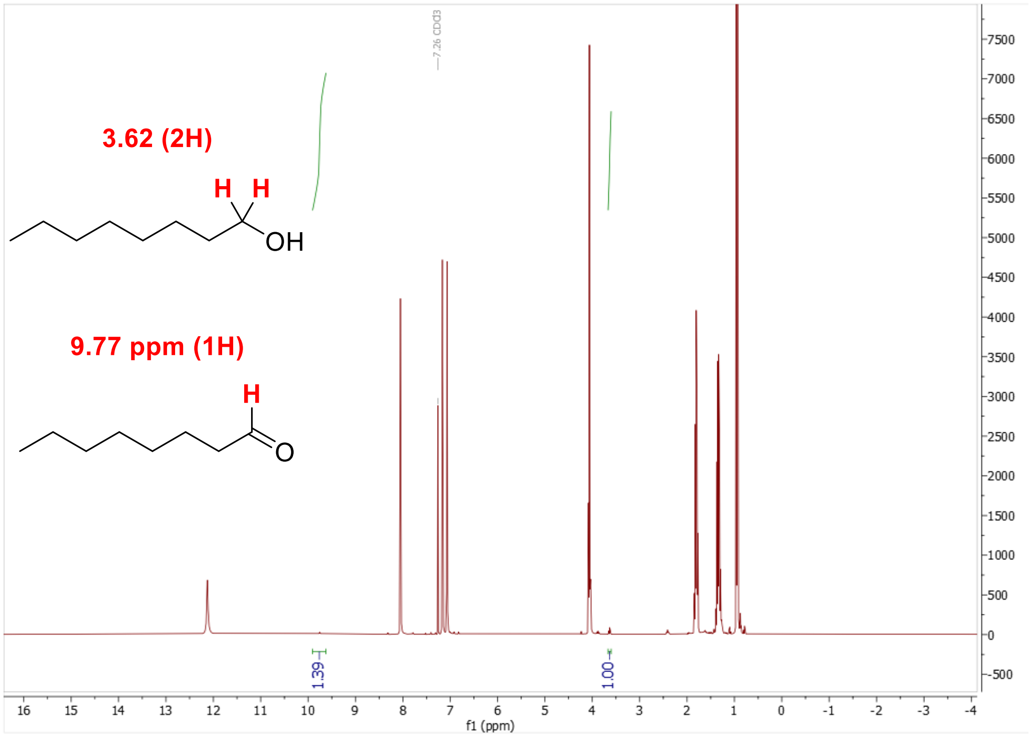

^{1}H NMR spectrum of Betulin, 13

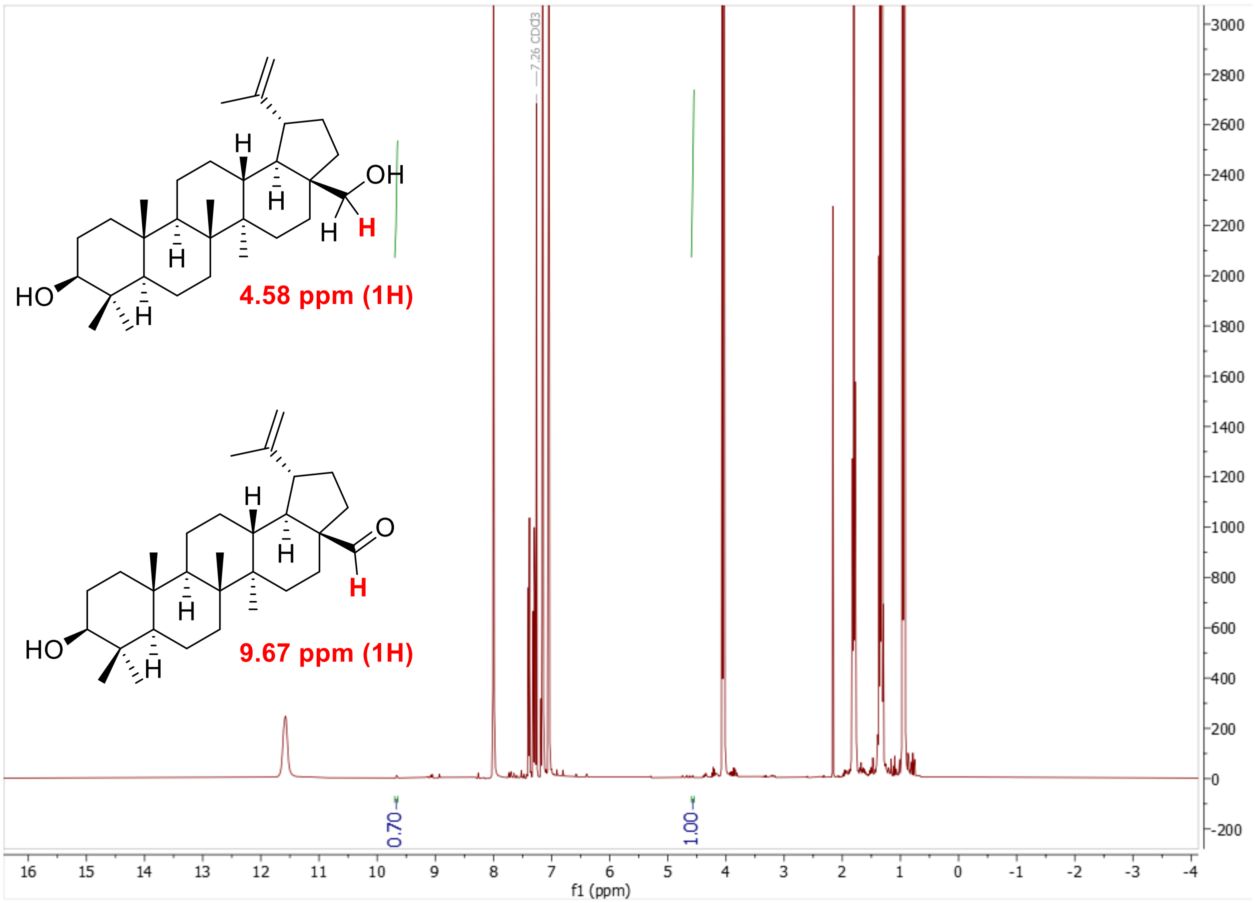

References

(1) Winsberg, J.; Stolze, C.; Schwenke, A.; Muench, S.; Hager, M. D.; Schubert, U. S. Aqueous 2,2,6,6-Tetramethylpiperidine-N-Oxyl Catholytes for a High-Capacity and High Current Density Oxygen-Insensitive Hybrid-Flow Battery. *ACS Energy Lett.* **2017**, *2* (2), 411–416. https://doi.org/10.1021/acsenergylett.6b00655.

(2) Cosier, J.; Glazer, A. M. A Nitrogen-Gas-Stream Cryostat for General X-Ray Diffraction Studies. *J. Appl. Crystallogr.* **1986**, *19* (2), 105–107. https://doi.org/https://doi.org/10.1107/S0021889886089835.

(3) CrysAlisPro Software System, Rigaku Oxford Diffraction. Rigaku Corporation, Oxford, UK 2018.

(4) Dolomanov, O. V; Bourhis, L. J.; Gildea, R. J.; Howard, J. A. K.; Puschmann, H. OLEX2: A Complete Structure Solution, Refinement and Analysis Program. *J. Appl. Crystallogr.* **2009**, *42* (2), 339–341. https://doi.org/10.1107/S0021889808042726.

(5) Sheldrick, G. M. SHELXT - Integrated Space-Group and Crystal-Structure Determination. *Acta Crystallogr. Sect. A* **2015**, *71* (1), 3–8. https://doi.org/10.1107/S2053273314026370.

(6) Green, R. A.; Brown, R. C. D.; Pletcher, D.; Harji, B. An Extended Channel Length Microflow Electrolysis Cell for Convenient Laboratory Synthesis. *Electrochem. Commun.* **2016**, *73*, 63–66. https://doi.org/10.1016/j.elecom.2016.11.004.

(7) Bini, R.; Chiappe, C.; Mestre, V. L.; Pomelli, C. S.; Welton, T. A Rationalization of the Solvent Effect on the Diels-Alder Reaction in Ionic Liquids Using Multiparameter Linear Solvation Energy Relationships. *Org. Biomol. Chem.* **2008**, *6* (14), 2522–2529. https://doi.org/10.1039/b802194e.
